# Repurposing Azacitidine and Carboplatin to Prime Immune Checkpoint Blockade–resistant Melanoma for Anti-PD-L1 Rechallenge

**DOI:** 10.1158/2767-9764.CRC-22-0128

**Published:** 2022-08-17

**Authors:** Andre van der Westhuizen, Megan Lyle, Moira C. Graves, Xiaoqiang Zhu, Jason W. H. Wong, Kerrie Cornall, Shu Ren, Leanna Pugliese, Richard Levy, Adeeb Majid, Ricardo E. Vilain, Nikola A. Bowden

**Affiliations:** 1 Hunter Medical Research Institute and School of Medicine and Public Health, College of Health, Medicine and Wellbeing, University of Newcastle, Callaghan, NSW, Australia.; 2 Department of Medical Oncology, Calvary Mater Hospital, Newcastle, NSW, Australia.; 3 Liz Plummer Cancer Centre, Cairns Hospital, Cairns, Queensland, Australia.; 4 School of Biomedical Sciences, Li Ka Shing Faculty of Medicine, University of Hong Kong, Hong Kong SAR, P.R. China.; 5 Department of Surgery, Calvary Mater Hospital, Newcastle, NSW, Australia.; 6 Department of Anatomical Pathology, Pathology North, NSW Health Pathology, Newcastle, NSW, Australia.

## Abstract

**Purpose:**

Drug repurposing offers the opportunity for chemotherapy to be used to reestablish sensitivity to immune checkpoint blockade (ICB) therapy. Here we investigated the clinical and translational aspects of an early phase II study of azacitidine and carboplatin priming for anti-PDL1 immunotherapy (avelumab) in patients with advanced ICB-resistant melanoma.

**Experimental Design:**

A total of 20 participants with ICB-resistant metastatic melanoma received 2 × 4-week cycles of azacitidine and carboplatin followed by ICB rechallenge with anti-PD-L1 avelumab. The primary objective was overall response rate after priming and ICB rechallenge. Secondary objectives were clinical benefit rate (CBR), progression-free survival (PFS), and overall survival (OS). Translational correlation analysis of HLA-A and PD-L1 expression, RNA sequencing, and reduced representation bisulfite sequencing of biopsies at baseline, after priming and after six cycles of avelmuab was performed.

**Results:**

The overall response rate (ORR) determined after azacitidine and carboplatin priming was 10% (2/20) with two partial responses (PR). The ORR determined after priming followed by six cycles of avelumab (week 22) was 10%, with 2 of 20 participants achieving immune partial response (iPR). The CBR for azacitidine and carboplatin priming was 65% (13/20) and after priming followed by six cycles of avelumab CBR was 35% (*n* = 7/20). The median PFS was 18.0 weeks [95% confidence interval (CI): 14.87–21.13 weeks] and the median OS was 47.86 weeks (95% CI: 9.67–86.06 weeks). Translational correlation analysis confirmed HLA-A generally increased after priming with azacitidine and carboplatin, particularly if it was absent at the start of treatment. Average methylation of CpGs across the HLA-A locus was decreased after priming and T cells, in particular CD8^+^, showed the greatest increase in infiltration.

**Conclusions:**

Priming with azacitidine and carboplatin can induce disease stabilization and resensitization to ICB for metastatic melanoma.

**Significance:**

There are limited treatments for melanoma once resistance to ICB occurs. Chemotherapy induces immune-related responses and may be repurposed to reinstate the response to ICB. This study provides the first evidence that chemotherapy can provide clinical benefit and increase OS for ICB-resistant melanoma.

## Introduction

Despite significant progress in the treatment of metastatic melanoma with molecularly targeted and immune checkpoint blockade (ICB) therapy in recent years, there remains limited effective treatment once resistance occurs. The mechanisms of resistance to ICB are being extensively explored, and recently the downregulation of HLA-A expression and other key immune pathways have been identified as a key component in the development of ICB resistance (1, 2).

Drug repurposing offers the opportunity to determine whether more affordable, off-patent, and FDA-approved chemotherapy agents can be used to increase immune response pathways and reestablish sensitivity to ICB immunotherapy. The mechanism of “priming” aims to reestablish immune response in ICB-resistant patients. Epigenetic and DNA-damaging chemotherapy have long been known to induce immune-related responses in solid tumors, but used together they have the potential to synergistically prime to reinstate the response to ICB.

DNA methylation plays an important role in cancer and commonly alters the expression of tumor suppressor genes in the absence of somatic mutations (3). In cutaneous melanoma, global hypomethylation increases DNA instability and local hypermethylation of promoter CpG islands can silence the expression of tumor suppressor genes (4). These changes lead to alterations in pathways, including cell-cycle regulation, cell signaling, transcription, DNA repair, and apoptosis (5).

DNA methylation provides an appealing target for drug repurposing to overcome ICB resistance due to the established and safe use of DNA hypomethylating agents such as 5-aza-5′deoxycytidine (decitabine) and azacitidine. DNA hypomethylating agents are cytosine analogs that bind to DNA methyltransferase 1 and inhibit the function of maintaining DNA methylation during cell division, leading to passive demethylation with replication (6). The inhibition of methylation restores the expression of silenced genes (7). Azacitidine is used primarily in the treatment of hematologic malignancies, including myelodysplastic syndrome and chronic myeloid leukemia but has since become a useful mechanism for studying the effect of methylation on gene expression (8). At high doses, both decitabine and azacitidine can be cytotoxic, inducing DNA damage from DNTM1 persistently bound to DNA, but at low doses, can effectively demethylate without causing cell death (9, 10).

Preclinical studies and clinical trials have investigated the therapeutic potential of DNA hypomethylating agents as monotherapy, and in combination, in several solid cancer types with positive results. Of the 16 most commonly used chemotherapy agents, DNA-damaging cisplatin and carboplatin show the best synergy with decitabine *in vitro* (11). This synergy affects transcriptional activation of silenced genes, with the addition of carboplatin increasing gene expression from a 10-fold increase to a 32-fold increase (11).

To date, the combination of hypomethylating and DNA-damaging agents has been reported in preclinical studies (12) but has not been assessed by clinical trials in melanoma. The combination has however, shown promising effects in ovarian cancer, with high response rate and progression-free survival (PFS) in platinum-resistant patients (13). Similar results have been found in head and neck squamous cell carcinoma (14), neuroblastoma (15), renal carcinoma (16), and non–small cell lung cancer (17).

Here we investigated the clinical and translational aspects of the “priming” combination of hypomethylating and DNA-damaging agents in an early phase II study of azacitidine and carboplatin priming for anti-PDL1 immunotherapy (avelumab) in patients with advanced melanoma who were resistant to ICB (PRIME002: ACTRN12618000053224).

## Materials and Methods

### Study Design

#### Sample Size Calculation

Power calculation was used to determine that the study was 80% powered (β = 0.2) to detect an overall response rate (ORR) of 20% (α = 0.05) for a sample size of 17. To ensure greater than 80% power was achieved, the study was closed with a sample size of 20.

### Research Subjects

This study was approved by the Northern Sydney Local Health District Human Research Ethics Committee, reference number HREC/17/HAWKE/55′ Australian clinical trial registry number: ACTRN12618000053224; registered January 16, 2018. Patients provided written informed consent in accordance with the Declaration of Helsinki, the Australian National Health and Medical Research Council's National Statement on Ethical Conduct in Research (2007), and the CMPH/ICH Note for Guidance on Good Clinical Practice.

A total of 20 patients with unresectable or metastatic melanoma with primary resistance to ICB immunotherapy were recruited to assess ORR, clinical benefit rate (CBR), and adverse events. All participants were on ICB for at least 3 months and had two timepoint imaging assessments (MRI brain and PET scan) 6–8 weeks apart to confirm disease progression before enrolment in the study. All participants receiving active treatment with ICB had confirmed disease progression as described above at the time of enrolment. Participants receiving BRAF and MEK inhibitor therapy after ICB were also confirmed to have resistance to targeted therapy before enrolled onto the study. All BRAF V600 positive participants were on > second-line therapy and had confirmed failure of prior targeted and ICB therapy. To ensure participants had true disease progression after induction therapy with combination immunotherapy of dual checkpoint blockade (anti-CTLA4 and anti-PD1 ICB), all participants completed partial (if they developed a significant immune-related toxicity) or full induction therapy as well as at least one or more cycles of maintenance therapy with anti-PD1 ICB before they were considered for the study.

The best response of all participants on their prior line of ICB therapy was partial response (PR)/stable disease (SD) or primary progressive disease (PD). No participants with complete response (CR) on previous line of ICB that later changed to SD/PD were enrolled onto the study as these patients were considered to have true secondary resistance. We aimed for a population of participants who demonstrated at least some form initial/primary resistance to ICB treatment which was demonstrated by an incomplete initial response to ICB treatment. Patients who stopped ICB treatment for a period of time because of CR/near CR/metabolic CR who then progressed or recurred at a later timepoint in the course of their disease were not recruited to participate in this study.

### Research Objectives

Primary Objective: Quantify ORR after two cycles of priming according to RECIST 1.1 and six cycles of immunotherapy (avelumab) according to iRECIST criteria (18).

Secondary Objectives: Quantify CR, PR, SD, and CBR after administration of two cycles of azacitidine and carboplatin/28-day cycle using RECIST 1.1; Quantify CR, PR, SD, and CBR after administration of two cycles of azacitidine and carboplatin/28-day cycle using RECIST 1.1 and six cycles of immunotherapy (avelumab) according to iRECIST; Determine genome-wide DNA methylation and transciptome changes after administration of two cycles of azacitidine and carboplatin and six cycles of immunotherapy (avelumab); Quantify immune-response markers (PDL-1, PD-1, CD4/CD8, and CD68) in blood before treatment, after two priming cycles (azacitidine and carboplatin) and after six immunotherapy (avelumab) cycles; Calculate PFS and overall survival (OS) at each RECIST data collection point (weeks −1, 9, and 22) and every 6 months until study completion.

#### Interventional Early Phase II Study Design

Patients were treated with an epigenetic and platinum chemotherapy priming program of 2 × 4-week cycles (Table 1). Followed by an immunotherapy maintenance program until disease progression, death, or withdrawal from study (Table 2).

**TABLE 1:** Epigenetic and platinum chemotherapy priming treatment schema

Week	−2	−1	1	2	5	6	8	9
Day	−14	−8	1–5	8	29–33	36	50–56	57–61
Treatment		RECIST	AZA 40 mg/m^2^ IVI/day	Carbo AUC 4.5 IVI	AZA 40 mg/m^2^ IVI/day	Carbo AUC 4.5 IVI	Subcut lesion Blood	RECIST
Sample and data collection	Subcut lesion Blood		Adverse events assess	Adverse events assess	Adverse events assess	Adverse events assess	Adverse events assess	

**TABLE 2:** Immunotherapy treatment schema

Week	10	12	14	16	18	20	22		24 onward
Day	64	78	92	106	120	134	148–152		162 onward
Treatment	Avelumab 10 mg/kg IVI	Avelumab 10 mg/kg IVI	Avelumab 10 mg/kg IVI	Avelumab 10 mg/kg IVI	Avelumab 10 mg/kg IVI	Avelumab 10 mg/kg IVI	Avelumab 10 mg/kg IVI		Avelumab 10 mg/kg/2 weeks until PD, toxicity or death
Sample and data collection	Blood+ Adverse events assess	Blood+ Adverse events assess	Blood+ Adverse events assess	Blood+ Adverse events assess	Blood+ Adverse events assess	Blood+ Adverse events assess	Subcut lesion Blood Adverse events assess	RECIST	CT scan every 12 weeks to monitor response Adverse events continuously assessed at every clinic visit/2 weeks

#### Primary Outcome Measures

ORR after two cycles of priming (1 cycle = azacitidine × 5 days followed by carboplatin on day 8/28-day cycle) according to RECIST 1.1 and six cycles (1 cycle = 1 dose of avelumab/14-day cycle) of immunotherapy according to iRECIST.

#### Secondary Outcome Measures

CR, PR, SD, and CBR after two cycles of priming (1 cycle = azacitidine × 5 days followed by carboplatin on day 8/28-day cycle) according to RECIST 1.1 and six cycles (1 cycle = 1 dose of avelumab/14-day cycle) of immunotherapy according to iRECIST.

#### Translational Experimental Design

##### Immune Cell Profiles

Whole blood was collected at baseline, week 9 (after two cycles of azacitidine and carboplatin) and week 22 (after six cycles of avelumab). Whole blood was separated into peripheral blood mononuclear cells (PBMC) and plasma and stored at −80°C.

PBMCs were thawed rapidly, washed in RPMI media, rested for 1 hour at 37°C, and washed twice more in RPMI. The cells were resuspended at a concentration of 1–10 × 10^6^ cells/mL, and 1 μL of BD Horizon Fixable Viability stain 575V was added and incubated at room temperature for 15 minutes. Samples were washed twice with BD Pharmingen Stain Buffer. The following targets were costained: Hu CD3 BUV737 UCHT1, Hu CD4 BUV496 SK3, Hu CD8 APC-H7 SK1, Hu CD279(PD-1) BB515 EH12.1, Hu TIM-3(CD366) Alexa 647 7D3, Hu LAG-3(CD223) APC-R700 T47-530 was added to each sample and incubated for 30 minutes at 2°C–8°C. The samples were washed twice with stain buffer. After final wash, 350 μL of stain buffer was added to each sample and data acquired on a Fortessa X20 (BD Biosciences). Each sample was analyzed in duplicate, nonconsecutively.

Gated T cells (CD3^+^, live, single cell, lymphocytes) were gated as CD4^+^ or CD8^+^ and further divided by presence of PD-1, LAG3, or TIM3. Coexpression of the three exhaustion markers was assessed but no further analysis was conducted because of very low percentage of cells positive for two or more exhaustion markers. Student two-tailed *t* test was used to compare the mean of each group and one-way ANOVA was performed with multiple comparison (Bonferroni) testing between the individual timepoints for each group in our cohort. Statistical analysis was performed on data using FlowJo v12, SPSS V25 and R.

#### Tumor Biopsies

Duplicate tumor biopsies were collected at baseline, after 2 × cycles of priming (week 9) and after six cycles of avelumab (week 22) if metastatic disease was amenable to biopsy. A total of 4 of 20 patients had metastatic disease that was amenable to biopsy by palpation or detected by CT at all three timepoints. A total of 16 of 20 patients did not have disease amenable to biopsy at all timepoints. Each of the duplicate biopsies were formalin-fixed, paraffin embedded (FFPE) for HLA-A expression analysis and fresh frozen for RNA sequencing (RNA-seq) and reduced representation bisulfite sequencing.

#### HLA-A and PD-L1 Expression

FFPE biopsies for 4 participants (001, 006, 008, 010) collected at baseline, week 9 and week 22 were confirmed to contain metastatic melanoma using hematoxylin and eosin (H&E)-stained sections by a pathologist (R.E. Vilain), with the exception of 001 week 9 biopsy which did not contain viable melanoma. FFPE tumor biopsies were sliced into 4 μm sections and processed for 3′,3′-diaminobenzidine (DAB) IHC using a Ventana Discovery Ultra (Roche) by the Hunter Cancer Biobank. Sections were labeled using recombinant Anti-HLA-A antibody (EP1395Y; AbCam) or Ventana PD-L1 (SP263) Assay (Roche). All steps from baking to chromogen addition were performed automatically by the Ventana Discovery Ultra. Tissue sections were baked to slides and deparaffinized, and antigen retrieval then occurred at 95°C/pH 9 with a total incubation time of 24 minutes prior to the addition of the primary antibody. Addition of the primary antibody was followed by a 32-minute incubation at 36°C. Slides were then incubated with secondary antibody Anti-Rabbit HQ (Roche) for 20 minutes at 36°C. Slides were digitally scanned using the Aperio Digital AT2 Pathology System (Leica Biosystems) for analysis.

HLA-A and PD-L1 assessment was performed by a pathologist blinded to all treatment timepoint and clinical information. Staining intensity was graded from 0 to 3 as described previously (19); 0 = complete absence, 1 = low intensity and/or <50% cells positive, 2 = medium intensity staining and >50% cells positive, 3 = high intensity staining and >50% cells positive. The small cohort (*n* = 4) only allowed for descriptive analysis of the results, statistical analysis could not be performed.

### RNA-seq

Frozen biopsies for 3 participants (006, 008, 010) collected at baseline, week 9 and week 22 were confirmed to contain metastatic melanoma using H&E-stained sections by a pathologist (R.E. Vilain), with the exception of patient 010 week 22 biopsy which did not contain viable melanoma. RNA was extracted from biopsies using the AllPrep DNA/RNA/Protein Mini Kit (Qiagen) as per manufacturer's instructions. RIN score was determined by High Sensitivity RNA Screen Tape analysis (Agilent) and RNA was quantified using Qubit HS. KAPA RNA HyperPrepKit with RiboErase was used for total RNA library prep with rRNA depletion and the eight RNA libraries were sequenced on NovaSeq. RNA-seq library prep and NovaSeq was provided by the Garvan Institute of Medical Research Kinghorn Centre for Clinical Genomics Sequencing Laboratory.

The RNA-seq data were aligned to hg38 using the STAR aligner (20). To quantify the expression of genes, featureCounts (21) was used to extract read counts for genes from GENCODE annotations. Gene expression was normalized across samples using the trimmed mean of M-values method (22). For the analysis of transposable element expression, the REdiscoverTE pipeline was used (23). Briefly, this pipeline uses Salmon (24) to quantify repetitive elements from RepeatMasker (25) that do not overlap with annotated genes and aggregates transposable element (TE) expression at the family and subfamily level.

#### Whole-genome Bisulfite Sequencing

As described for RNA-seq, frozen biopsies for 3 participants (006, 008, 010) collected at baseline, week 9 and week 22 were confirmed to contain metastatic melanoma, with the exception of patient 010 week 22 biopsy which did not contain viable melanoma. DNA was extracted from biopsies using the AllPrep DNA/RNA/Protein Mini Kit (Qiagen) as per manufacturer's instructions. DNA was quantified using Qubit HS (Invitrogen). Eight DNA libraries were bisulfite converted and sequenced on HiSeq by the Garvan Institute of Medical Research Kinghorn Centre for Clinical Genomics Sequencing Laboratory.

The whole-genome bisulfite sequencing (WGBS) data were analyzed using the Bismark pipeline (26) against hg38. CpG island annotations were obtained from the UCSC table browser. For gene annotations, CpGs were assigned to a gene within 1 kb of its transcription start site, and the final methylation value was the averaged. For the methylation of TE, CpGs that overlapped with RepeatMasker TEs were averaged to obtain methylation values for each TE family.

#### Flow Cytometry

Immune cell subsets from peripheral blood collection at baseline, after 2 × cycles of azacitidine and carboplatin (week 9) and after six cycles of avelumab (week 22) were determined using Flow Cytometry on the BD Science (Becton, Dickinson and Company) Fortessa X20 as described previously (27, 28). Frozen PBMCs were thawed and stained with the following targets: horizon Fixable Viability stain 575V, Hu CD3 BUV737 UCHT1, Hu CD4 BUV496 SK3, Hu CD8 APC-H7 SK1, Hu CD279(PD-1) BB515 EH12.1, Hu TIM-3(CD366) Alexa 647 7D3, Hu LAG-3(CD223) APC-R700 T47-530. Samples were gated to acquire 50,000 live cell events and for lymphocytes, single cells and then live cells. The CD3 subset was gated as the fluorophore (BUV737) versus side scatter (SSE). To separate into CD4^+^ and CD8^+^ subsets, all positive CD3^+^ cells were then further divided into CD4^+^ and CD8^+^ by gating CD3 versus CD4^+^ (BUV496) or CD8^+^ (APC-H7 SK1). For each of the other surface markers (e.g., TIM3), positive CD4 or CD8 were gated against the other surface markers, e.g., CD4^+^ (BUV496 SK3) versus TIM-3 (Alexa 647 7D3). Statistical analysis of immune cell subsets was performed using R and SPSS. A *P* value of <0.05 was considered statistically significant. For multiple testing corrections, Benjamini–Hochberg FDR was calculated.

### Data Availability

Data are available upon request from the European Genome-phenome Archive (EGA) RNA-seq ID: EGAS00001006419 and WGBS ID: EGAS00001006420.

## Results

### Cohort Characteristics

Participants with metastatic melanoma (*n* = 20) were recruited between March 2018 and December 2020. All participants had confirmed disease progression whilst treated with single-agent anti-PD1 ICB (*n* = 12) or combination anti-CTLA4 and anti-PD1 ICB (*n* = 8). A total of 8 participants had also received targeted BRAF/MEK inhibitors prior to ICB. The average number of lines of previous treatment was 2, with a range of 1–8 lines.

The average age was 63.9 ± 14.4 years and the cohort consisted of 4 females and 16 males. Eastern Cooperative Oncology Group (ECOG) scores were all 0 or 1, with the exception of ECOG score 2 for participant 017. A summary of the cohort characteristics is presented in Table 3.

**TABLE 3:** Cohort characteristics

Patient characteristics	*n* (%)
Age (years), median (range)	64 (40.0–86.0)
**Sex**	
Male	16 (80)
Female	4 (20)
**Metastatic site**	
Liver	7 (35)
Bone	7 (35)
Brain	5 (25)
**Baseline LDH expression**	
≤ULN	13 (65)
> ULN - ≤ 2 × ULN	6 (30)
> 2× ULN	1 (5)
**Prior lines of treatment**	
1	7 (35)
2	6 (30)
3	2 (10)
>3	5 (25)
**Prior anti PD-1 therapy**	
Pembrolizumab	14 (70)
Nivolumab	6 (30)
Ipilimumab	6 (30)
PD-1 and CTLA 4 combination	8 (40)
**Prior targeted therapy**	
Dab + Trem	7 (35)
Vem + Cabi	4 (20)
Enco + Bini	2 (10)
**BRAF status**	
V600 mutation	8 (40)
Wild type	12 (60)
**ECOG performance status**	
0	10 (50)
1	7 (35)
2	2 (10)
Unknown	1 (5)

### ORR

The primary outcome measure of the study was the ORR after two cycles of priming according to RECIST 1.1 (29, 30) and then after six cycles of ICB (avelumab) rechallenge according to iRECIST (18). The ORR of epigenetic and DNA-damaging “priming” treatment was determined after 2 × 4-week cycles of azacitidine and carboplatin priming (week 9) was 10% (2/20) with two PRs. No CRs occurred.

The ORR of epigenetic and DNA-damaging priming followed by immunotherapy rechallenge determined after 2 × 4-week cycles of azacitidine and carboplatin priming followed by six cycles of anti-PD-L1 immunotherapy (avelumab; week 22) was 10%, with 2 of 20 participants achieving iPR. Interestingly, the 2 participants with PR after azacitidine and carboplatin priming did not maintain the PR during immunotherapy rechallenge. The 2 participants with iPR after six cycles of avelumab had SD after priming with responses close to PR at −28.1% and −29.7%.

### CBR

The secondary outcome measure of the study was CBR determined as the percentage of participants with CR, PR, or SD after 2 × 4-week cycles of azacitidine and carboplatin priming (week 9) and then after six cycles of immunotherapy (avelumab) rechallenge. In addition to the 10% (2/20) PR, 55% (11/20) maintained SD resulting in a CBR for azacitidine and carboplatin priming of 65% (13/20).

The CBR of epigenetic and DNA-damaging priming followed by immunotherapy rechallenge was determined after 2 × 4-week cycles of azacitidine and carboplatin priming followed by six cycles of anti-PD-L1 immunotherapy (avelumab) according to iRECIST. In addition to the 10% iPR (2/20), 25% (5/20) maintained SD resulting in CBR of 35% (*n* = 7/20).

#### Tumor Response to Epigenetic and DNA-damage Priming for Immunotherapy Rechallenge

**FIGURE 1:**
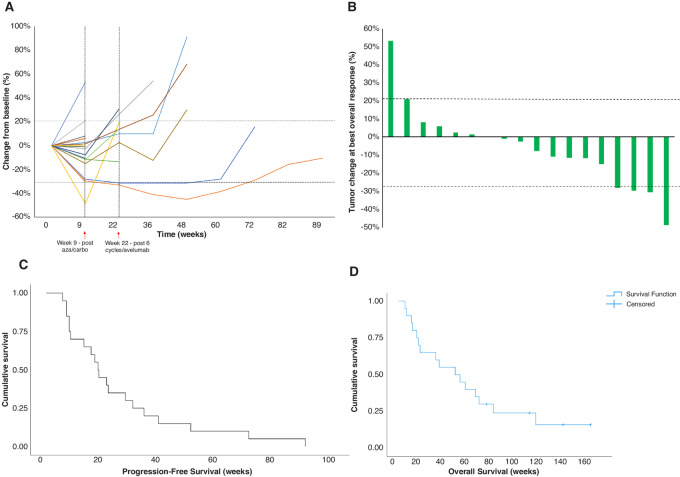
Tumor response, PFS, and OS. **A,** Spider plot of tumor burden change of target lesions in reference to the baseline tumor burden in 18 patients with measurable tumor burden. RECIST/iRECIST, biopsies, and translational bloods were collected at week 9 after azacitidine/carboplatin and week 22 after six cycles of avelumab (indicated by dashed lines). **B,** Waterfall plot of the tumor burden change of target lesions at best response (%) in reference to the baseline tumor burden in 18 patients with measurable tumor burden. Dashed lines at +20% and −30% represent the threshold used for progression (PD) and PR. **C,** PFS in weeks. The median PFS from start of treatment was 18.0 weeks (95% CI: 14.87–21.13 weeks). **D,** OS in weeks. The median OS was 47.86 weeks (95% CI: 9.67–86.06 weeks).

Disease stabilization or PR was achieved for RECIST 1.1 measurable target lesions for 94.4% (17/18) patients that completed 2 × 4-week cycles of epigenetic and carboplatin priming (Fig. 1A). The best overall response was PR or SD for 90% (18/20) patients (Fig. 1B).

A total of 2 patients rapidly progressed and did not complete the two cycles of azacitidine and carboplatin priming. A total of 3 patients with stable target lesions had confirmed PD according to RECIST 1.1 due to unequivocal progression of nontarget lesions (*n* = 2) or a new lesion (*n* = 1).

Disease stabilization or PR was achieved for iRECIST measurable target lesions for 77.8% (7/9) patients that completed 2 × 4-week cycles of epigenetic and carboplatin priming followed by six cycles of avelumab (Fig. 1A). A total of 55% (11/20) patients had confirmed PD before week 22.

### PFS and OS

The median PFS from the start of cycle 1 of azacitidine and carboplatin was 18.0 weeks [95% confidence interval (CI): 14.87–21.13 weeks] (Fig. 1C). The proportion of PFS at week 9 after two cycles of priming was 65% (13/20) of participants, which decreased to 35% (7/20) by week 22 (Table 4).

**TABLE 4:** PFS and OS

	Azacitidine and carboplatin priming (week 9)	Avelumab six cycles (week 22)	Median weeks (range)
**PFS**	13/20 (65%)	7/20 (35%)	18.0 (5.71–90.14)
**OS**	18/20 (90%)	13/20 (65%)	47.86 (5.71–161.29)

The median OS from the start of cycle 1 was 47.86 weeks (95% CI: 9.67–86.06 weeks; Fig. 1D). A total of 90% (18/20) of participants survived beyond week 9 and 65% (13/20) beyond week 22 (Table 4).

#### Assessment of Safety

No grade 4 or persistent grade 3 treatment-related adverse events (TRAE) occurred as result of the azacitidine and carboplatin priming or rechallenge with avelumab. All reported grade 1–3 TRAEs are summarized in Table 5.

**TABLE 5:** TRAEs

CTCAE Grade	TRAE	Count	Patients (*n*)	Patients (%)
Grade 4		0	0	0
Grade 3	Neutropenia^a^	2	2	10%
	Infusion reaction^a^	1	1	5%
	Pneumontis^a^	1	1	5%
	Fever^a^	1	1	5%
Grade 2	Mucositis	1	1	5%
	Infusion reaction	1	1	5%
	Rash	1	1	5%
	Neutropenia	1	1	5%
	Pneumonitis	1	1	5%
	Diarrhea/Colitis	1	1	5%
	Fevers	1	1	5%
	urinary tract infection	1	1	5%
	Fatigue	1	1	5%
Grade 1	Neutropenia	4	4	20%
	Fatigue	4	4	20%
	Nausea	4	4	20%
	Lethargy	3	2	15%
	Dysgeusia	4	3	15%
	constipation	2	2	10%
	Headaches	2	2	10%
	Hepatitis	2	2	10%
	Decreased Lymphocytes/white cell count	2	1	5%
	anaemia	1	1	5%
	Hypomagnesemia	1	1	5%
	Joint symptoms	1	1	5%
	loose stools	1	1	5%
	Metallic taste- dysgeasia	1	1	5%
	Platelet Count Decreased	1	1	5%
	vomiting	1	1	5%
	weight loss	1	1	5%
	Diarrhea/ Colitis	1	1	5%
	Rash	1	1	5%

^a^Duration <7 days.

Whole blood counts were collected at baseline, week 9 (after two cycles of azacitidine and carboplatin) and week 22 (after six cycles of avelumab). There was no significant difference in whole blood cell counts at week 9 or week 22 compared with baseline (Supplementary Fig. S1).

#### Translational Aspects of Priming Mechanism and Immunotherapy Rechallenge: Expression of HLA-A and PD-L1 After Epigenetic and Carboplatin Priming and Immunotherapy Rechallenge

**FIGURE 2:**
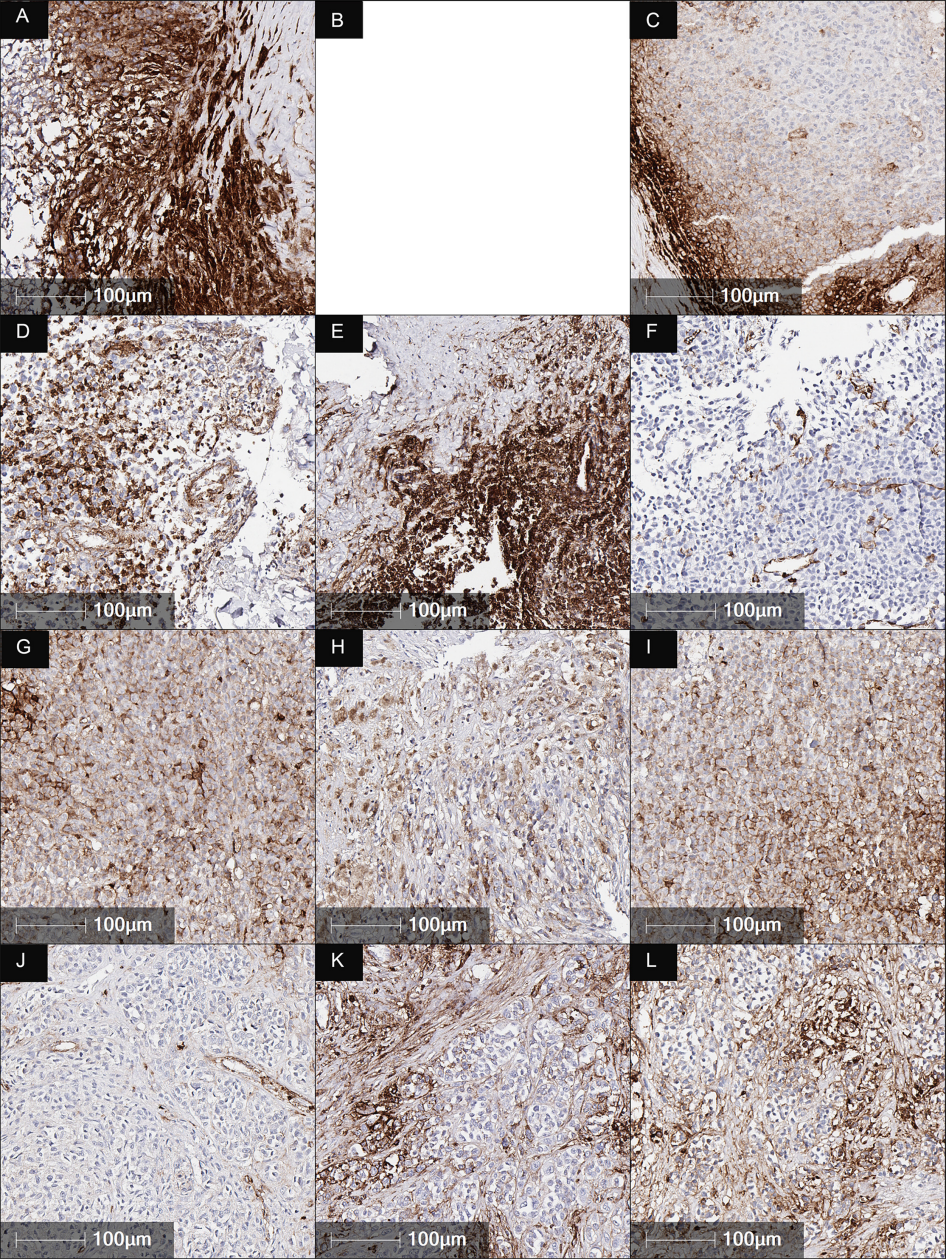
HLA-A IHC in baseline (**A**, **D**, **G**, **J**), week 9 (**B**, **E**, **H**, **K**) and week 22 (**C**, **F**, **I**, **L**) biopsies. HLA-A generally increased after priming with azacitidine and carboplatin and rechallenge with avelumab, particularly if it was absent at the start of treatment.

Biopsies were limited to 4 participants as tissue collection was restricted to accessible nodal or subcutaneous lesions and where the lowest risk of morbidity was expected in doing the biopsy. Despite the small sample size, HLA-A and PD-L1 expression and lymphocyte infiltrate was investigated at baseline, after priming (week 9) and after ICB rechallenge (week 22). HLA-A generally increased after priming with azacitidine and carboplatin and rechallenge with avelumab in comparison with baseline biopsies, particularly if it was absent at the start of treatment (Fig. 2). The increased HLA-A expression was seen in areas of immune cell populations and was predominantly in tumor cells at the immune interface (Fig. 2H, I, K, and L).

**FIGURE 3:**
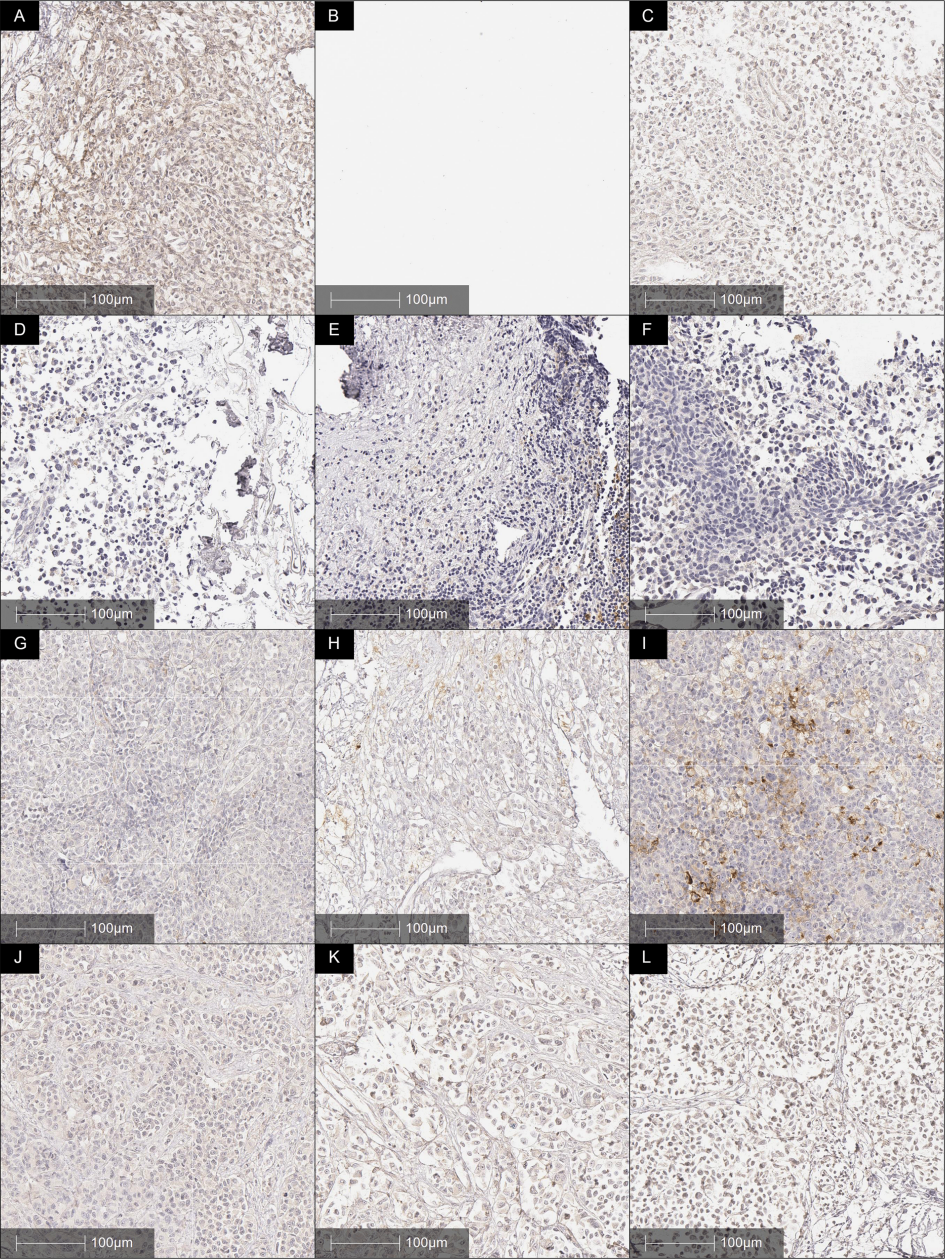
PD-L1 IHC in baseline baseline (**A**, **D**, **G**, **J**), week 9 (**B**, **E**, **H**, **K**) and week 22 (**C**, **F**, **I**, **L**) biopsies. PD-L1 was negative at baseline, after priming with azacitidine and carboplatin and rechallenge with avelumab with the exception of one biopsy at week 22 which has 1%–2% PD-L1–positive tumor and immune cells (**C**).

All biopsies at all timepoints were negative for PD-L1 (Fig. 3) except one biopsy at week 22 (Fig. 3I) that displayed islands of PD-L1–positive staining with small clusters of PD-L1–positive tumor and immune cells. 1%–2% of tumor was positive for PD-L1.

#### Altered DNA Methylation and Gene Expression of Antigen Presentation and Immune Signaling After Epigenetic and Carboplatin Priming and Immunotherapy Rechallenge

Epigentic (WGBS) and transcriptomic (RNA-seq) profiling were performed for 3 patients (Pt 6, Pt 8, and Pt 10). These patients all had SD at week 9, and at week 22, biopsies were measured as SD by iRECIST, but Pt 8 and 10 had one new lesion at week 22 and were iCPD. Frozen biopsies were available for all timepoints for the three patients except at week 22 for Pt 10.

**FIGURE 4:**
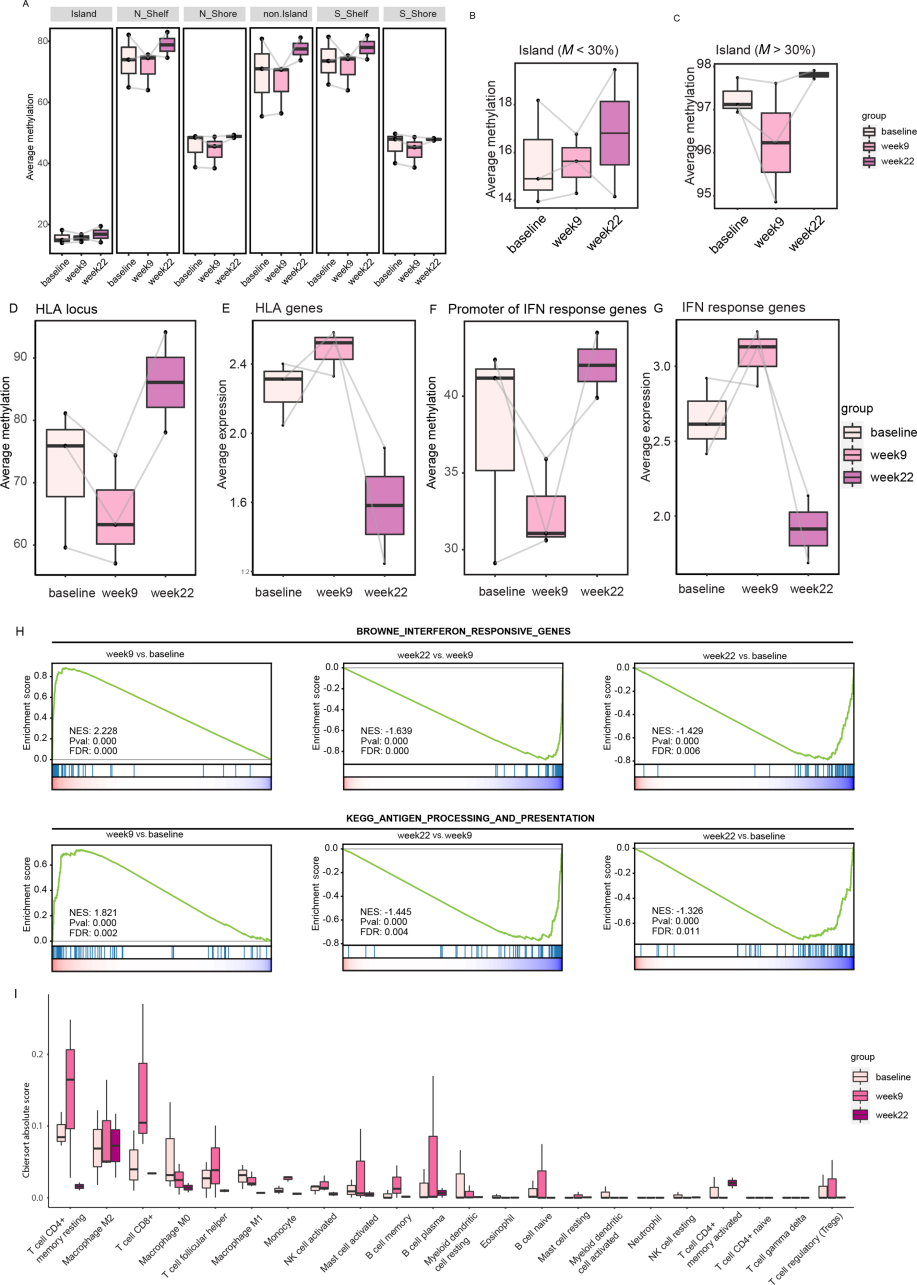
DNA methylation and gene expression analysis of patient samples at baseline, week 9 and week 22. Average methylation across different CpG loci in relation to CpG islands (**A**). Average methylation of unmethylated (**B**) and methylated (**C**) CpG islands. Average CpG methylation (**D**) and gene expression (**E**) change across HLA-A locus. Average CpG methylation change at IFN response gene promoters (**F**) and gene expression change of corresponding IFN response genes (**G**). Geneset enrichment analysis of IFN response and antigen processing and presentation pathway for timepoint comparisons (**H**). CIBERSORT scores of immune cell infiltration at the different timepoints (**I**).

We first assessed the impact of genome-wide DNA methylation changes induced by azacitidine treatment. CpGs were stratified in relation to CpG islands. As expected, most CpG islands are unmethylated, with shores partially methylated. CpG island shelves and non-island CpGs were mostly methylated (Fig. 4A). Upon treatment, at week 9, there was a modest change in methylation level across all regions, with no noticeable trend for Pt 6 and Pt 10 across week 9 and week 22, while for Pt 8, there was a general decrease at week 9 and increase at week 22. Comparing unmethylated (< 30%; Fig. 4B) and methylated (>30%; Fig. 4C) CpG islands, it was found that decrease in methylation level at week 9 was consistent across all patients, suggesting that azacitidine has the greatest effect on DNA methylation at hypermethylated CpG islands.

As IHC indicated an increase in HLA-A staining following priming (week 9; Fig. 2), we sought to determine whether this was related to DNA demethylation caused by azacitidine treatment. Using the WGBS data, we quantified the average methylation of CpGs across the HLA-A locus, which indeed showed a consistent decrease in methylation at week 9 in all 3 patients (Fig. 4D). This demethylation was mirrored by an increase in the expression of HLA-A genes at week 9 (Fig. 4E).

To further assess the impact of azacitidine priming on immune response, we also assessed the impact of DNA demethylation on IFN response genes. Two patients (Pt 6 and Pt 8) showed substantial average demethylation of the promoters of IFN response genes, while there was a slight increase in Pt 10 (Fig. 4F). The impact of the change in DNA methylation was again reflected in the average expression of these genes (Fig. 4G). More generally, using the gene expression data for gene set enrichment analysis, we found a significant increase in the expression of IFN response and antigen processing and presentation pathways (Fig. 4H) when comparing week 9 with baseline. Interestingly, this trend is reversed by week 22, which may reflect a change in the composition of both the tumor and its microenvironment. These results are intriguing, but the small cohort size (*n* = 3) limited our ability to draw conclusions from the data. Further validation of the results is required in a study with a larger cohort of participants and biopsies.

Recent studies have shown that one of the mechanisms of action of azacitidine treatment is to induce the expression of TE (31). Using the REdiscoverTE pipeline (23), we quantified changes in TE across the three timepoints. Interestingly, unlike IFN and antigen presentation, there was a sustained increase in the expression of most TE subfamilies even at week 22. However, this did not corroborate with average methylation change at these TE loci, which suggested that TE overexpression may be restricted to limited loci, making it difficult to detect by average methylation levels (Supplementary Fig. S2).

Finally, we used CIBERSORT (32) to determine the level of infiltration of different immune cells in these tumors. At baseline, T cells and macrophages had the highest CIBERSORT scores suggesting that they are the most dominant infiltrating immune cells in these tumors. Importantly, at week 9, it was found that T cells, in particular CD8^+^, showed the greatest increase in infiltration (Fig. 4I). CD8^+^ T cells can indicate a “hot” tumor microenvironment and is a predictor of immunotherapy response (32). This is consistent with our hypothesis that azacitidine and carboplatin prime the tumor microenvironment for response to ICB therapy.

#### T-Cell Markers in Peripheral Blood After Epigenetic and Carboplatin Priming and Immunotherapy Rechallenge

**FIGURE 5:**
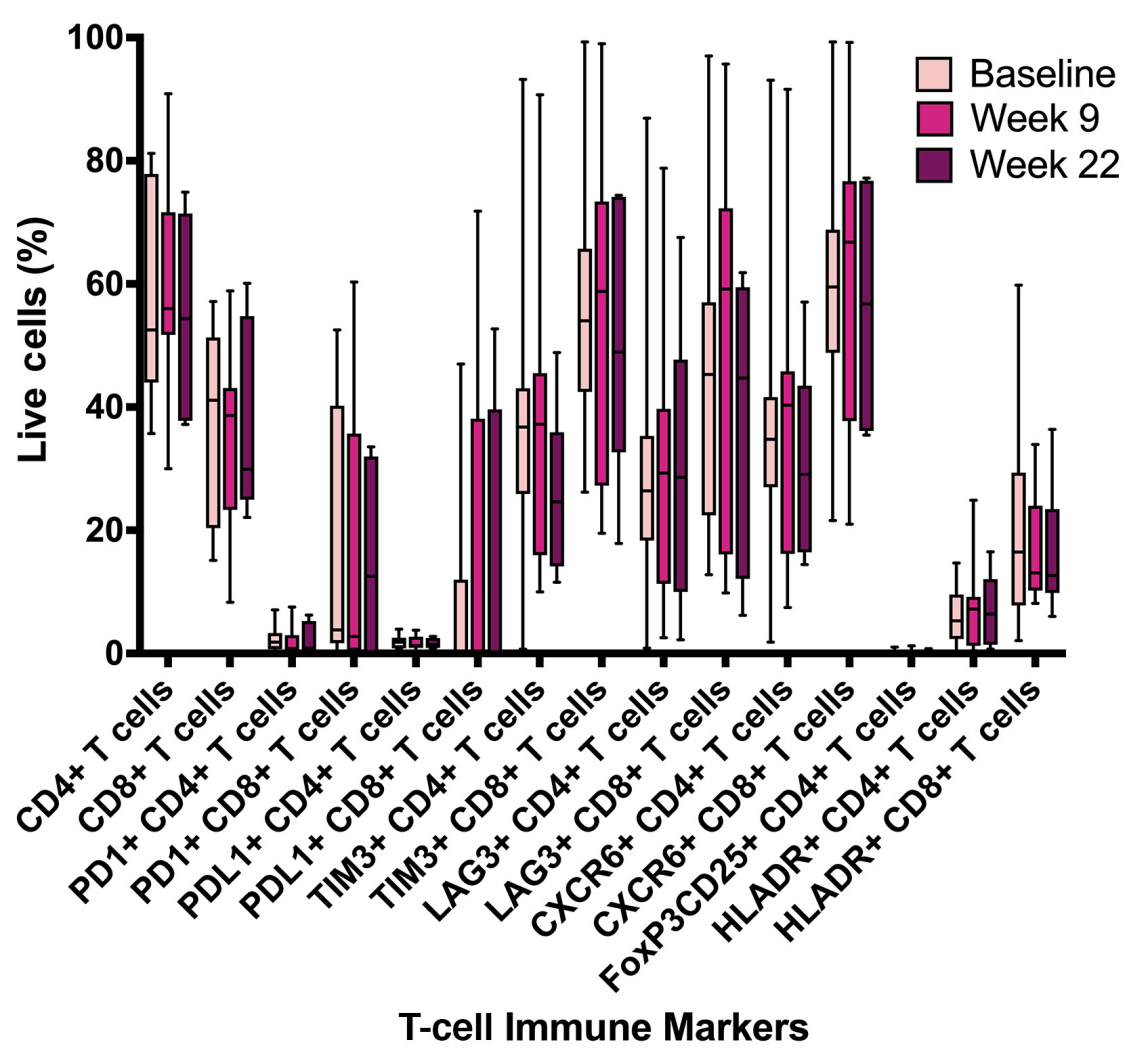
T-cell exhaustion proliferation and migration markers. Flow cytometry was used to quantify markers from peripheral blood at baseline, week 9 after two cycles of azacitidine and carboplatin and week 22 after six cycles of avelumab.

T-cell exhaustion proliferation and migration markers were quantified from peripheral blood using flow cytometry at baseline, week 9 after two cycles of azacitidine and carboplatin and week 22 after six cycles of avelumab. There was no significant difference in the markers at week 9 or week 22 compared with baseline. There was an overall trend of a small increase after priming at week 9 which requires confirmation in a larger cohort (Fig. 5).

## Discussion

The clinical and translational data in this early phase II study suggest that the mechanism of priming with azactidine and carboplatin changes the tumor dynamics by enhancing the pathways related to antigen expression resulting in stabilization of disease burden and response to ICB rechallenge. The regime was well tolerated as indicated by the low incidence and grade of toxicities (described in Table 5) suggesting that priming does not need to be at MTD to be beneficial. Participants were enrolled onto the study soon after progression was confirmed on their previous line of therapy with two imaging modalities (both PET and MRI brain) 4–8 weeks apart. Because of the unknown safety profile of azacitidine and carboplatin in advanced melanoma, patients were only considered for the study if they continued to have low ECOG performance status and adequate organ function relative to their burden of disease at the point of the second set of scans when progression was confirmed. The priming phase stabilized disease burden in the majority of participants (75%) with minimal toxicity and most maintained the low ECOG status to safely enter ICB rechallenge at week 9. The established safety profile will allow further studies to determine the CBR for azacitidine and carboplatin in a cohort with poor ECOG performance status to understanding if the priming regime is beneficial for high ECOG performance.

This study design consisted of two cycles of priming with azacitidine and carboplatin which resulted in SD for 65% (13/20) of patients and PR in a further 10% of patients (2/20). The low ECOG status of the cohort may have contributed to the 75% CBR but there was no direct relationship between baseline lactate dehydrogenase (LDH) and SD or PR, indicating that high burden of disease may not be a limiting factor in the potential use of the priming regime. The data suggest that more cycles of priming in future studies may need to be considered to improve clinical responses, antigen presentation, and expression of immune-related transcripts. The results are intriguing, but require follow-up in a larger cohort with higher ECOG performance status.

HLA-A encodes for an antigen-presenting MHC class I molecule. HLA-A complexes with beta-2 microglobulin to present viral and tumor-derived peptides on antigen-presenting cells for recognition by alpha-beta T-cell receptor on CD8^+^ T cells, guiding antigen-specific T-cell immune response to eliminate cells. Increasing the expression of HLA-A may therefore overcome ICB resistance and allow previously resistant patients to be rechallenged with ICIs. The clinical problem we face is how to increase HLA-A expression in ICB-resistant melanoma.

The highest level of antigen pathway expression occurred during or shortly after priming but returned to the same or lower levels by six cycles of avelumab. The increase in antigen pathway expression may need to be captured by combining some or all of the priming cycles with ICB in future studies. The data also suggest that there is loss of expression of the antigen pathway over time which indicates that repriming at 4–6 months intervals may be beneficial to maintain sensitivity to ICB.

Betof Warner and colleagues (33) recently reported an ORR of 14.7% (5/34) after of reinduction of single-agent anti-PD-1 in patients that had previously been treated with ICB. In contrast to the study described herein, the patient population reported by Betof Warner and colleagues (33) was not selected for confirmed ICB resistance, 27% (21/78) of retreated patients discontinued initial ICB treatment due to toxicity or suspected CR. Of the 5 patients who responded to retreatment with single-agent anti-PD-1, 2 patients initially discontinued because of toxicity. The conclusions of the study stated that “response rates to retreatment with checkpoint inhibitor (CPI) therapy after relapse from single-agent anti-PD-1 were disappointingly low; future studies should seek to determine alternative strategies to overcome resistance.” The authors also noted “several mechanisms of resistance to CPIs have been proposed, including adaptive immune resistance and phenotypic changes of residual tumor cells, such as changes in HLA class I, antigen processing or presentation, or programmed death-ligand-1 expression.” Therefore, the preliminary results of priming with azacitidine and carboplatin presented here describe an alternative strategy to overcome resistance that is worthy of further investigation.

The most convincing support for further investigation was the 75% PFS rate at week 9 (after two cycles of azacitidine and carboplatin). The decline in response to 35% PFS at week 22 after six cycles of single-agent anti-PD-L1 ICB suggest that increasing the number of priming cycles, overlapping with commencement of ICB or and the use of anti-PD1 or combination anti-CTLA4/anti-PD1 ICB may be beneficial. It is unknown whether anti-PD1 ICB would respond to azacitidine and carboplatin priming better than the anti-PD-L1 ICB avelumab did in this study. The absence of PD-L1 expression in biopsies in this study indicates it would be beneficial to investigate alternate ICB to take advantage of the increased antigen presentation pathways occurring as a result of priming. Further studies to assess anti-PD1 and/or anti-CTLA4 ICB after azacitidine/carboplatin priming are required to answer this question. Rechallenge with combination anti-CTLA4 and anti-PD1 after priming may be most beneficial to improve responses in future studies, especially considering the impact of priming on HLA expression in this study and the mechanism of action of anti-CTLA4 ICB (34).

The data suggest that other platinum-resistant tumor groups may benefit from the priming mechanism to establish or reestablish ICB sensitivity. A recent study in high-grade serous ovarian cancer also investigated the combination of the DNA methyltransferase inhibitor guadecitabine and concluded that inhibiting DNA methylation altered gene expression related to DNA repair and immune activation and resensitized to carboplatin. There is high potential to further exploit the response to sensitize ovarian cancer to ICB (35, 36).

In conclusion, priming with azacitidine and carboplatin can induce disease stabilization and resensitization to ICB for metastatic melanoma. Future larger prospective clinical and translational studies are needed to further elucidate the role and mechanism of priming as a way to improve or reestablish responses to ICB.

## Supplementary Material

Supplementary Figure 1 Supplementary Figure 1 shows Whole blood cell counts at baseline, week 9 and week 22

Supplementary Figure 2 Supplementary Figure 2 shows transposable element methylation (A) and expression (B) at baseline, week 9 and week 22
