# Supplementary figures and images for: Repurposing Azacitidine and Carboplatin to Prime Immune Checkpoint Blockade–resistant Melanoma for Anti-PD-L1 Rechallenge
Source: Cancer Res Commun. 2022 Aug 17;2(8):814–26. doi: 10.1158/2767-9764.CRC-22-0128 (PMC10010343; doi:10.1158/2767-9764.CRC-22-0128)

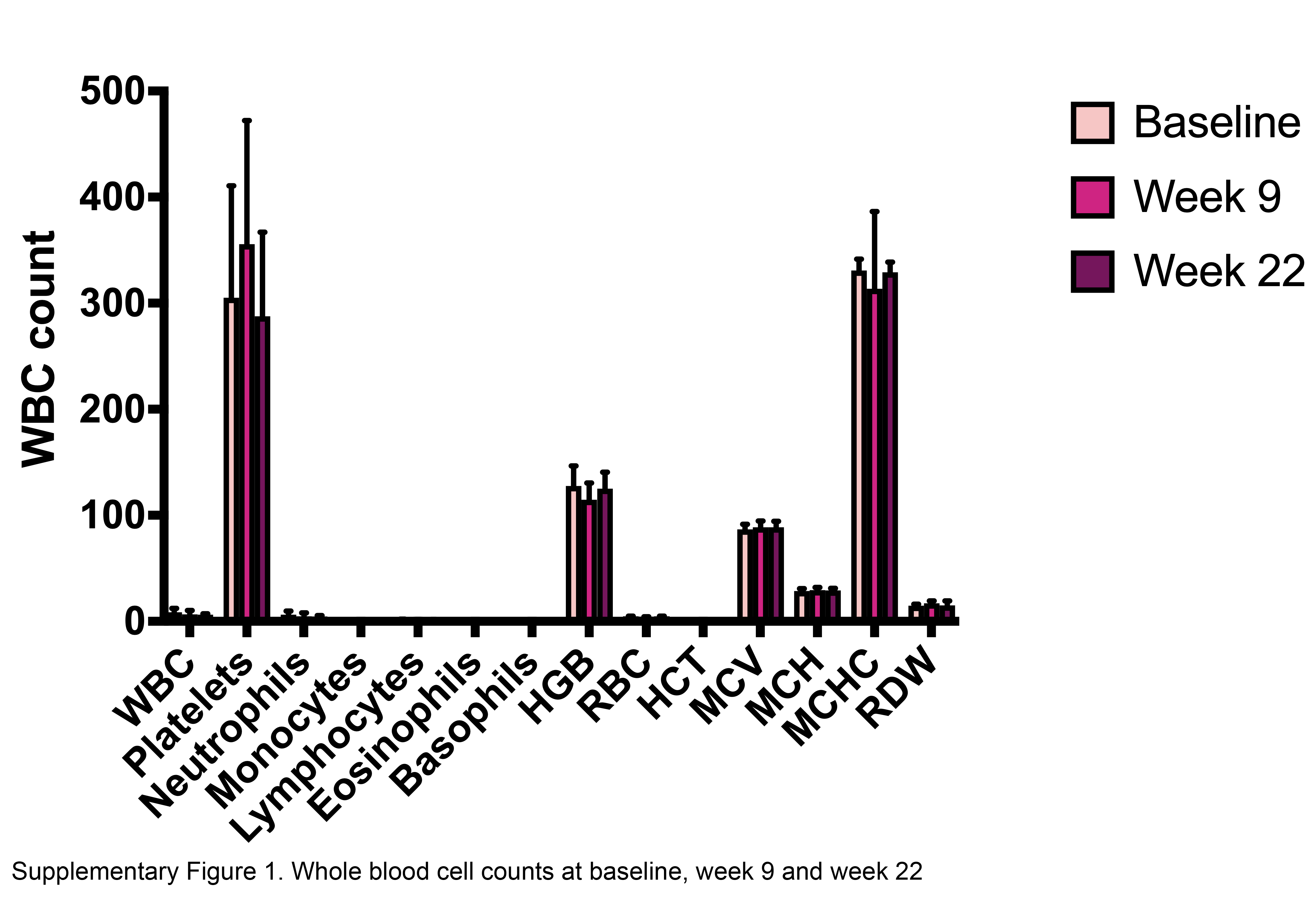

Supplement: Supplementary Figure 1 — shows Whole blood cell counts at baseline, week 9 and week 22 [file crc-22-0128-s01.png]

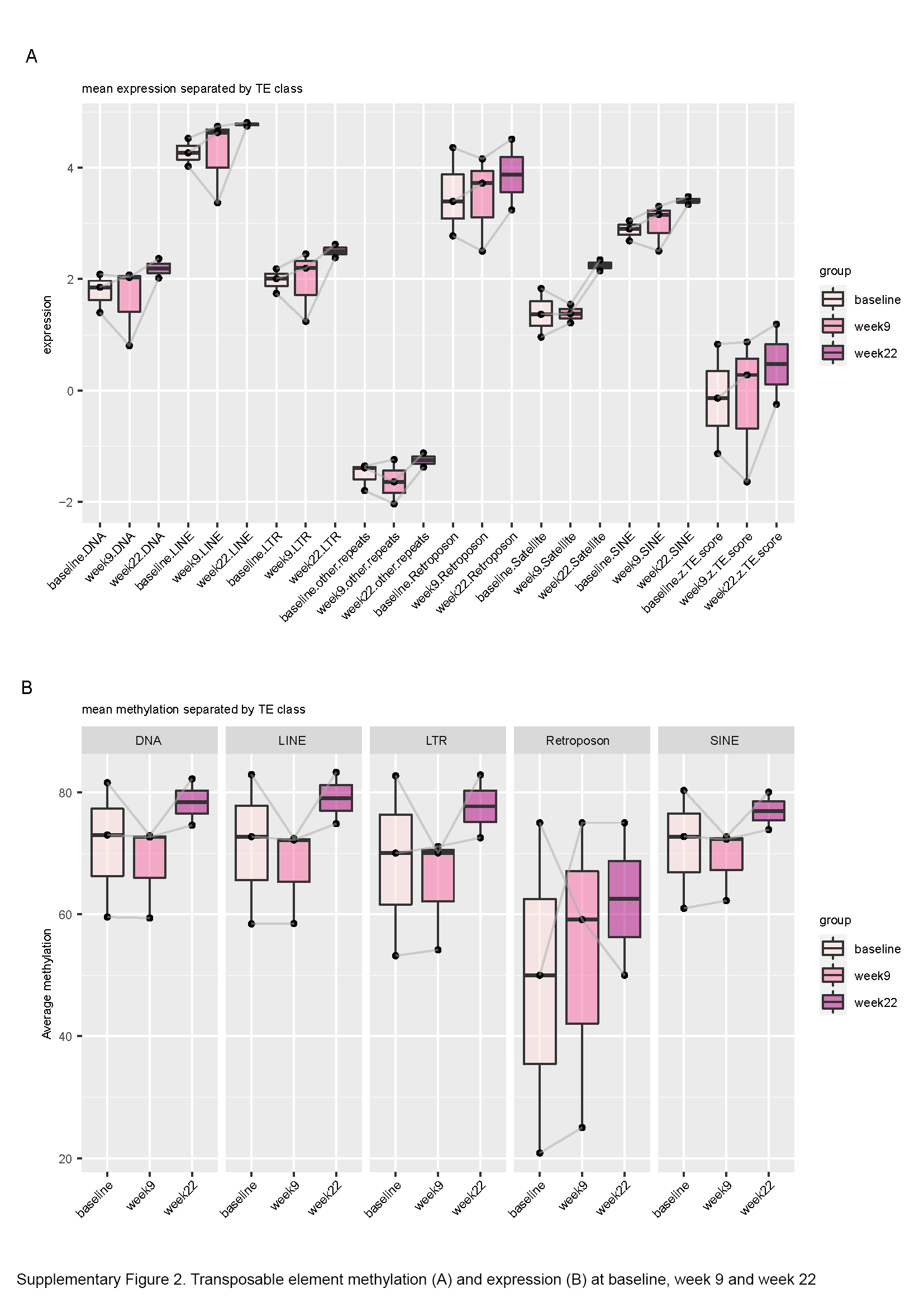

Supplement: Supplementary Figure 2 — shows transposable element methylation (A) and expression (B) at baseline, week 9 and week 22 [file crc-22-0128-s02.png]
